# A Single-Institution Review of Portosystemic Shunts in Children: *An Ongoing Discussion*

**DOI:** 10.1155/2010/964597

**Published:** 2010-05-06

**Authors:** J. B. Lillegard, A. M. Hanna, T. J. McKenzie, C. R. Moir, M. B. Ishitani, D. M. Nagorney

**Affiliations:** Divisions of Pediatric, GI, and General Surgery, Mayo Clinic, Rochester, 200 First Street, South west, Rochester, MN 55905, USA

## Abstract

*Purpose*. Review the safety and long-term success with portosystemic shunts in children at a single institution. *Methods*. An IRB-approved, retrospective chart review of all children ages 19 and undergoing surgical portosystemic shunt from January 1990–September 2008. *Results*. Ten patients were identified, 8 females and 2 males, with a mean age of 15 years (range 5–19 years). Primary diagnoses were congenital hepatic fibrosis (5), hepatic vein thrombosis (2), portal vein thrombosis (2), and cystic fibrosis (1). Primary indications were repeated variceal bleeding (6), symptomatic hypersplenism (2), and significant liver dysfunction (2). Procedures performed were distal splenorenal bypass (4), side-to-side portocaval shunt (3), proximal splenorenal shunt (2), and an interposition H-graft portocaval shunt (1). There was no perioperative mortality and only minor morbidity. Seventy percent of patients had improvement of their symptoms. Eighty percent of shunts remained patent. Two were occluded at a median follow-up of 50 months (range 0.5–13.16 years). Two patients underwent subsequent liver transplantation. Two patients died at 0.5 and 12.8 years postoperatively, one from multisystem failure with cystic fibrosis and one from post-operative transplant complications. *Conclusions*. The need for portosystemic shunts in children is rare. However, in the era of liver transplantation, portosystemic shunts in selected patients with well-preserved liver function remains important. We conclude that portosystemic shunts are safe and efficacious in the control of variceal hemorrhage and symptoms related to hypersplenism.

## 1. Introduction

The approach to portal hypertension (PH) in children has evolved significantly over the past half-century. With improved endoscopic treatments for variceal bleeding, liver transplantation and transjugular intrahepatic portosystemic shunts (TIPSs), the use of portal systemic shunts (PSSs) has been relegated to a few selected patients in the pediatric population. However, our experience and the experience of others [1–7] continue to support the valuable role of PSSs in selected children.

PH in children can be divided into two main categories: (i) extrahepatic portal hypertension (EHPH) and (ii) intrahepatic portal hypertension (IHPH). EHPH in children is commonly due to main portal vein thrombosis, which may variably extend into the splenic, mesenteric, or intrahepatic portal veins. In the setting of EHPH, liver function is typically preserved. Consequently, hepatic decompensation is rare and liver transplantation is seldom indicated. In contrast, in patients with IHPH, hepatic decompensation often occurs over time as evident clinically by ascites, coagulopathy, and encephalopathy. IHPH in children can result from a number of distinct causes including congenital hepatic fibrosis, hepatic vein thrombosis, inborn errors of metabolism, biliary atresia, and cystic fibrosis.

Esophagogastric variceal hemorrhage, splenomegaly, and hypersplenism are the common clinical manifestations of both EHPH and IHPH. Historically, repeated variceal hemorrhage has been the primary indication for PSSs in children [7, 8]. Although PSSs have clearly proven efficacious in controlling variceal hemorrhage, persistent concerns regarding variable degrees of hepatic encephalopathy and its effects have persisted [2, 9]. Yet recent reports of PSSs have not substantiated those outcomes [3, 5]. Indeed, patients with patent grafts have had good long-term health and no decrease in scholastic performance related to occult portal-systemic encephalopathy (PSE) [1, 3, 5, 10]. 

 Our study aims to further assess the outcomes of PSSs in children to determine whether PSSs in patients with EHPH or IHPH are clinically valuable in the era of liver transplantation.

## 2. Methods

### 2.1. Patient Selection

This study was approved by the IRB at the Mayo Clinic, Rochester. Ten pediatric patients who underwent PSSs were identified retrospectively between 1990 and 2008 through our computer database. All patients during this time period were included in this report regardless of the causes leading to surgical PSSs. 

There were 8 females and 2 males with a mean age of 15 years (range 5–19 years). Patients were followed for 6 months to 13 years with a median followup of 50 months. Our electronic database was examined for preoperative and postoperative (1) demographic findings, (2) liver and renal functions, (3) serum ammonia levels, (4) ascites, (5) type of shunt performed, (6) primary and secondary diseases leading to portal hypertension, (7) indication for surgical PSS, (8) 30-day operative mortality, (9) shunt patency, (10) intra-operative complications, (11) patient survival, and (12) subsequent need for liver transplantation (Table 1). Model for End-stage Liver Disease (MELD) scores were calculated using serum creatinine (mg/dL), bilirubin (mg/dL) and INR values collected just before surgery and at various times postoperatively. The MELD score is calculated using the following formula: MELD = (0.957 × log_e_ (serum creatinine mg/dL) + 0.378 × log_e_ (serum bilirubin mg/dL) + 1.120 × log_e_ (INR) + 0.643) 10. All MELD scores were rounded to the nearest whole number. If a patient received two or more dialysis treatments within a given week where scores were generated, then serum creatinine levels were set at 4 mg/dL.

Primary indications for PSSs were repeated variceal bleeding despite endoscopic therapy (6), symptomatic hypersplenism with thrombocytopenia (2), and liver dysfunction in the setting of hepatic vein thrombosis (2). Evaluation of portal hypertension was conducted by Doppler ultrasonography of the portal venous system and hepatic veins. In addition, endoscopic evaluation of esophageal and gastric varices was conducted on all patients preoperatively. Liver biopsies were performed on patients with fibrotic liver disease. All patients undergoing PSSs had pre- and postoperative liver and renal functions testing. Selected patients had MR venography of the portal venous system, renal veins, and IVC to assess the degree of patency. Visceral arteriogram and hepatic venograms were also used selectively to evaluate patency and size of abdominal vasculature.

### 2.2. Surgical Techniques

**Figure 1:**
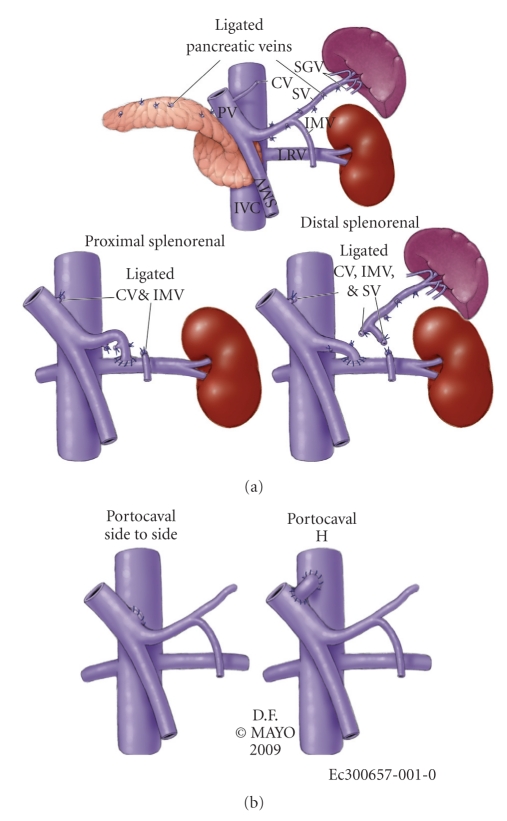
The four types of shunts used in our series. PV: portal vein; CV: cardiac vein; SV: splenic vein; IMV: inferior mesenteric vein; LRV: left renal vein; SMV: superior mesenteric vein; IVC: inferior vena cava; SGV: short gastric veins.

 PSSs performed were distal splenorenal bypass in 4, side-to-side portocaval shunt in 3, proximal splenorenal shunt in 2, and a portocaval H-graft using a spiral vein graft in one patient (Figure 1).

## 3. Results

Primary diagnoses were congenital hepatic fibrosis (5), hepatic vein thrombosis (2), portal vein thrombosis (2), and cystic fibrosis (1) (Table 1). Patients in this study were grouped into two categories based on the causes of their portal hypertension (Table 1). Those patients with portal vein thrombosis were categorized as EHPH, and all other patients with congenital hepatic fibrosis, hepatic vein thrombosis, and cystic fibrosis with subsequent fibrotic liver disease were categorized as IHPH. Only three patients with IHPH had liver dysfunction as evident by an elevated MELD score.

 Six patients underwent PSSs for repeated variceal bleeding despite endoscopic therapy. Both patients with portal vein thrombosis had variceal bleeding as the primary indication for PSSs. One of these patients is alive and well 7 years after a DSRS, which remains patent. The other patient, who had an orthotopic liver transplant as an infant, developed a portal vein thrombosis, which was complicated by variceal bleeding 14 years after liver transplantation. The PSRS thrombosed one year postoperatively and that patient is under evaluation for revisional PSS. 

The remaining 4 patients with variceal bleeding as the main reason for PSSs had primary diagnoses of congenital fibrosis (3) and cystic fibrosis (1). Two patients with congenital hepatic fibrosis are alive and well with patent shunts at 7.4 and 1 years postoperatively. The remaining patient with congenital hepatic fibrosis, who had significant preoperative liver dysfunction (MELD = 20), had an early postoperative variceal hemorrhage after PSS. Repeat operation failed to confirm shunt thrombosis. Despite nonexistence of further variceal hemorrhage, the patient underwent liver transplantation 13 months later and shunt patency was confirmed. The patient with cystic fibrosis died with a patent shunt 6 months after shunting due to multisystem failure as a complication of cystic fibrosis. 

Two patients underwent PSSs due to significant liver dysfunction resulting from hepatic venous thrombosis. One patient who underwent a side-to-side portocaval shunt had progression of the hepatic venous thrombosis to include thrombosis of the retrohepatic IVC and thrombosis of the shunt three months postoperatively. This patient underwent liver transplantation but died 12 years later from hepatorenal failure awaiting a second liver transplantation. The other patient with hepatic vein thrombosis and significant liver dysfunction received a portocaval H-graft shunt using a spiral vein graft. This patient is alive and well 13 years postoperatively with a patent shunt.

The final two patients with IHPH underwent PSSs for symptomatic hypersplenism, which included symptomatic splenomegaly and thrombocytopenia. The first patient underwent a DSRS. One patient that underwent DSRS sustained a significant reduction in splenomegaly and normalization of his platelet count and has confirmed shunt patency 8 years postoperatively. The second patient underwent a side-to-side portocaval shunt and also sustained a similar reduction in splenomegaly and normalization of leukocyte count along with a partial correction of his thrombocytopenia (63–86 × 10^9^ platelets per liter). This patient has a confirmed patent shunt 2 years postoperatively. Therefore, both patients with symptomatic hypersplenism received significant long-term relief of their symptoms following their shunt procedures and neither patient required a splenectomy.

**Figure 2:**
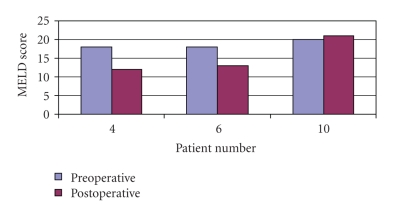
MELD/PELD scores of the patients with significant liver dysfunction. Significant liver dysfunction in this study was set at a MELD/PELD score of 15. Three patients (patients 4, 6, and 10) had significant liver dysfunction before shunt. Two of the three patients saw a significant improvement in liver function post shunt.

In our practice we performed 10 open PSSs in the time period listed. Three of these patients had liver dysfunction as measured by their MELD scores. However, 2 of these 3 patients received a significant improvement in their liver function following their shunt procedure and one of these patients has been able to maintain this improvement at long-term followup (13 years) (Figure 2). 

To assess for potential PSE after PSSs, the followup records and questionnaires were examined in 8 of 10 patients. Two patients were excluded because they lacked specific followup regarding PSE (patients number 5 and 7). Four patients noted an increase in fatigue and a decrease in scholastic performance and or short-term memory (Table 2). There was no association between PSE and serum ammonia levels.

## 4. Discussion

 Our findings confirm the safety and efficacy of elective PSS in selected children for complications of portal hypertension. Although our experience was small, this data supports similar reports from others [1–7]. PSSs provided durable control of variceal hemorrhage and symptomatic hypersplenism and was not associated with rapid deterioration of liver function or clinical encephalopathy. These findings suggest that PSSs should remain a treatment option for the complications of portal hypertension in selected children in the era of liver transplantation.

Multiple previous publications about PSSs in children or involving children have shown PSSs to be safe and effective with excellent long-term patency rates. There were no operative deaths in our series and operative and perioperative complications were limited. The patency rate of 80% in our patients is similar to that of others [3, 5, 7] and led to durable relief of the portal hypertensive indications for operation. Admittedly PSSs in our patients were performed both selectively and electively. PSS was performed infrequently over the period of study, which reflects the general trend for PSSs nationally. Although three of our patients had impaired liver function, selection of children for PSSs was based on failure of medical intervention and preserved liver function. In fact, two of our patients had EHPH and those with IHPH had underlying hepatopathies in which liver function is typically maintained over many years. All PSSs herein were performed electively. We did not encounter any patient who required emergent PSS for persistent variceal hemorrhage. This finding likely resulted from improved management of patients with acute variceal hemorrhage pharmacologically, endoscopically, and intravascularly and the location of our referral practice. 

Selection of PSSs was dependent anatomically upon the degree of patency of the portal venous system, size of the portal venous branch for shunting, and underlying liver function. Selection of PSSs in children was also affected by the goals of maintaining splenic function and minimizing the risk of encephalopathy or learning disorders. Preoperative imaging was performed to assess the degree of patency of the portal venous system and the absolute size of the major portal venous tributaries, which were relevant to the construction of the portosystemic venous anastomosis. Although portal venography was used in our early experience, dimensional imaging with contrast-enhanced computed tomography or magnetic resonance imaging with vascular reconstruction is now preferred because they accurately depict venous size and spatial relationships of the veins within the abdomen and the extent of thrombosis if present. In general, portal venous branch size of nearly 1 cm was preferred for elective PSS to reduce the likelihood of shunt thrombosis. Selective PSSs were preferred in patients with underlying chronic liver disease to maintain liver perfusion. 

Many of the treatment options for symptomatic hypersplenism in the setting IHPH presented in the literature for patients with well-preserved liver function involve some type of splenorenal shunt and splenectomy [4, 6, 7]. In the pediatric patient population, where long-term survival is often anticipated, a splenectomy comes with significant risks and problems making preservation of the spleen highly desirable [4, 6]. In our series spleen sparing central PSS was preferred to reduce the risk of postsplenectomy sepsis, which resulted in durable patent shunts and long-term cessation of preoperative symptoms. 

 In our patients with hepatic venous outflow obstruction (HVOO), central or nonselective PSSs were used to decompress the liver. Notably central PSSs for HVOO are often associated with durable improvement in liver function with relief of hepatic congestion [5, 7, 8]. Liver transplantation was clearly an alternative treatment for our patients with HVOO. Indeed, TIPS as a bridge to liver transplantation was certainly an emergent option for all patients with IHPH who were acutely bleeding, but such treatment was not required. TIPS has been performed successfully for patients with HVOO in a number of series, however, with low short-term patency rates ranging from 12%–60% necessitating additional interventions [11–13]. One patient in our series with HVOO was able to maintain the significant improvements in liver function achieved with the surgical PSS and has avoided the need for subsequent liver transplantation for 13 years with a patent shunt. This type of long-term patency is rarely achieved with the TIPS procedure in patients with HVOO [11–15]. 

Choice of PSS for EHPH was primarily dependent upon the extent of the portal venous thrombosis. Although the term “portal venous thrombosis” is used to denote any extent of thrombosis involving the portal venous system, defining the site and extent of thrombosis is essential to determine whether a PSS can be undertaken and which PSS can be constructed. We have limited PSSs to patients with thrombosis of the main portal vein with or without intrahepatic extension with patency of the superior mesenteric and splenic vein confluence. Isolated superior mesenteric vein patency does permit mesocaval shunting for variceal hemorrhage though concurrent splenectomy would be required for symptomatic hypersplenism. Isolated splenic vein thrombosis was not encountered though distal splenorenal shunting remains an option for such patients. We have had no experience with the “Rex Shunt”, which effectively is a portoportal or mesentericoportal venous bypass of an isolated segmental thrombosis of the main portal vein. Few publications exist regarding outcomes using the Rex Shunt; however, opponents of this shunt argue that it is associated with increased complications including decreased patency rates and an increased need for revisions [3]. Proponents of the Rex Shunt suggest that this shunt is more physiologic, is associated with decreased PSE, and improved somatic growth [16–19]. These same proponents suggest that the differences in complication rates between the Rex Shunt and other types of shunts, selective or non-selective, are associated with technique rather than the intrinsic factors of the shunt. 

There are potential long-term complications that have been reported in patients with PSSs. The most significant of these complications include shunt thrombosis, an increase risk of surgery during subsequent liver transplant, and PSE. Shunt thrombosis occurred in 2 patients (1 EHPH and 1 IHPH) in our series. The IHPH patient with a thrombosed shunt required a subsequent liver transplant and the EHPH patient has not had any subsequent variceal hemorrhage and is under consideration for a revisional shunt. Addressing thrombosed shunts in the pediatric population has not been well described. In some instances where thrombosis has occurred, subsequent evaluation shows the patient to be unshuntable and subsequent liver transplant and/or radical esophagogastrectomy are the only options. Some have described a salvage shunt that can be performed such as a mesocaval or mesogonadal shunt [20, 21]. However, these salvage shunts have been shown to be less durable than if they were performed as the initial shunt of choice.

Some of our patients were candidates for liver transplantation before PSSs. PSS has not precluded liver transplantation in our experience to date. Although liver transplantation clearly has advantages over PSS because it fully addresses the underlying liver disease when present, liver transplantation is associated with the need for lifelong immunosuppression, significant mortality and morbidity, and other long-term risks. 

The effects of PSSs and the development of PSE are still disputed. It is difficult to evaluate the effect of PSS and the development of PSE- related symptoms in the pediatric population due to the lack of adequate control groups. In a large prospective evaluation of PSSs in patients with EHPH, Orloff and colleagues made a thorough effort to evaluate PSE using the four-test index at each postoperative clinic visit. By their assessment of 200 patients at long-term follow-up no patient developed PSE [5]. In our series, screening for PSE- related symptoms involved questions regarding scholastic performance, fatigue, and perceived changes in memory. Screening was conducted at post-operative visits or by report from primary care providers near the patient's home (Table 2). In our study, 2 patients had EHPH, and at long-term followup neither patient has developed any symptoms related to PSE. Of interest, while patient number 2 did not experience a rise in serum ammonia levels, patient number 1 experienced a doubling of his serum ammonia level from 59 to 114 *μ*mol/liter. The reason this patient did not develop any symptoms related to PSE despite the increase in serum ammonia is likely due to the patient's well-preserved liver function pre- and postoperatively. Other studies have suggested that PSE symptoms do occur in some EHPH and IHPH patients who receive a PSS [2, 9]. Resolution of the difference in observation might be made by the example provided in our series. IHPH patients in our study were the only patients to report any PSE- related symptoms despite nonexistence of significant change in serum ammonia levels. It may be that patients with intrahepatic disease and or injury, even those whose liver function appears normal by traditional classifications, are still at higher risk for the development of PSE because at some level their livers are not able to cope with the changes created by the PSS. This appears to be the case in our series where symptoms attributed to PSE appeared to develop in 4 of 6 IHPH patients who were evaluated for PSE at followup (Table 2). Again of note, each one of the IHPH patients who developed PSE did not have an appreciable increase in his serum ammonia levels postoperatively and patients number 3 and 9 did not have any significant liver dysfunction before or after their shunt procedures.

Our series of patients is relatively small leading to what we see as the biggest criticism in which many of the lessons learned may be perceived as anecdotal. However, several important issues are raised in our report that can contribute to the overall discussion of PSSs in children. Our experience with PSSs in the pediatric population supports what is previously known in the literature in which patients with good liver function will likely have long-term durable results following their shunt procedures. Additionally, we provide support for a spleen preserving procedure for hypersplenism in the setting of IHPH. Most of these patients in the pediatric population are likely to have a long-term life expectancy and spleen-preservation should be obtained if possible. Patients, who have HVOO, pose a significant problem, as they will likely have a rapid deterioration of liver function and require immediate intervention. With regards to decompression, it is difficult to know whether TIPS or open PSS is best for these patients. Both TIPSs and open PSSs have been used as a short-term bridge to transplantation. However, our experience has shown that some patients who receive a surgical PSS may receive long-term decompression of their outflow obstruction with a patent shunt and avoid the need for transplantation. These long-term results appear to be directly attributable to the increased durability of open PSSs versus TIPSs. Finally, it appears from our series of patients that PSE after shunting represents an ongoing problem, but only in those patients with IHPH or liver injury regardless of the measured liver function or serum ammonia levels. Clearly these results raise the need for further studies into the measured components of PSE.

## Figures and Tables

**Table 1:** Ten patients were identified in the study. They are listed from 1 to 10 and referenced this way throughout the paper. DSRS: distal splenorenal shunt; PSRS: proximal splenorenal shunt; PCS: portocaval shunt; PV: portal vein; UTI: urinary tract infection. *MELD score 1 month post shunt. **MELD score 1 month and 13 years post shunt.

Patient no.	Age	Primary disease	Shunt type	MELD/PELD score pre- and postop		Primary reason for shunt	Complications	30-day mortality	Graft patency	Followup years
EHPH group										

1	17	PV Thrombosis	DSRS			Variceal Bleeding	None	None	Patent	7.08 Alive

2	17	PV Thrombosis	PSRS			Variceal Bleeding	None	None	Thrombosed	1.08 Alive

IHPH group										

3	17	Congenital Hepatic Fibrosis	DSRS	6	6	Variceal Bleeding	None	None	Patent	7.4 Alive

4	5	Congenital Hepatic Fibrosis	Side-to-side PCS	7	8	Variceal Bleeding	UTI, central-line infection	None	Patent	0.92 Alive

5	19	Cystic Fibrosis	DSRS	7	11	Variceal Bleeding	None	None	Patent	0.5 Died

6	15	Congenital Hepatic Fibrosis	PSRS	20	21	Variceal Bleeding	Bleeding Fundal Varices 10 days postop	Negative reexploration	Received liver transplant 13 months later post PSS	1.08 Alive

7	18	Hepatic Vein Thrombosis	Side-to-side PCS	18	12*	Liver Dysfunction	None	None	Shunt occluded at 3 months. Received liver txp 6 months later	12.8 Died

8	18	Hepatic Vein Thrombosis	PCSH-graft	18	13**	Liver Dysfunction	None	None	Patent	13.16 Alive

9	15	Congenital Hepatic Fibrosis	DSRS	7	7	Hypersplenism	None	None	Patent	6.5 Alive

10	10	Congenital Hepatic Fibrosis	Side-to-side PCS	8	11	Hypersplenism	None	None	Patent	1.92 Alive

**Table 2:** Survey of PSE symptoms along with ammonia levels pre and post PSS.

Patient	Ammonia Pre/Post PSS *μ*mol/liter		PSE Symptoms
1	59	114	None
2	20	16	None
3	19	31	Present
4	132	113	Present
6	59	68	Present
8	13	30	None
9	18	21	Present
10	11	30	None

## References

[1] Atta HM, Henderson JM, Galloway JR, Millikan WJ (1991). Selective splenocaval shunt: report of 26 cases and review of the literature. *Archives of Surgery*.

[2] Belli L, Puttini M, Marni A (1980). Extrahepatic portal obstruction. Clinical experience and surgical treatment in 105 patients. *Journal of Cardiovascular Surgery*.

[3] Botha JF, Campos BD, Grant WJ (2004). Portosystemic shunts in children: a 15-year experience. *Journal of the American College of Surgeons*.

[4] Hase R, Hirano S, Kondo S, Okushiba S, Morikawa T, Katoh H (2005). Long-term efficacy of distal splenorenal shunt with splenopancreatic and gastric disconnection for esophagogastric varices in patients with idiopathic portal hypertension. *World Journal of Surgery*.

[5] Orloff MJ, Orloff MS, Girard B, Orloff SL (2002). Bleeding esophagogastric varices from extrahepatic portal hypertension: 40 years’ experience with portal-systemic shunt. *Journal of the American College of Surgeons*.

[6] Robberecht E, Van Biervliet S, Vanrentergem K, Kerremans I (2006). Outcome of total splenectomy with portosystemic shunt for massive splenomegaly and variceal bleeding in cystic fibrosis. *Journal of Pediatric Surgery*.

[7] Wolff M, Hirner A (2003). Current state of portosystemic shunt surgery. *Langenbeck’s Archives of Surgery*.

[8] Fonkalsrud EW (1990). Treatment of variceal hemorrhage in children. *Surgical Clinics of North America*.

[9] Thompson EN, Williams R, Sherlock S (1964). Liver function in extrahepatic portal hypertension. *The Lancet*.

[10] Alagille D, Carlier J-C, Chiva M, Ziadé R, Ziadé M, Moy F (1986). Long-term neuropsychological outcome in children undergoing portal-systemic shunts for portal vein obstruction without liver disease. *Journal of Pediatric Gastroenterology and Nutrition*.

[11] Bureau C, Pagan JCG, Layrargues GP (2007). Patency of stents covered with polytetrafluoroethylene in patients treated by transjugular intrahepatic portosystemic shunts: long-term results of a randomized multicentre study. *Liver International*.

[12] Corso R, Intotero M, Solcia M, Castoldi MC, Rampoldi A (2008). Treatment of Budd-Chiari syndrome with transjugular intrahepatic portosystemic shunt (TIPS). *Radiologia Medica*.

[13] Murad SD, Luong TK, Pattynama PMT, Hansen BE, van Buuren HR, Janssen HLA (2008). Long-term outcome of a covered vs. uncovered transjugular intrahepatic portosystemic shunt in Budd-Chiari syndrome. *Liver International*.

[14] Gandini R, Konda D, Simonetti G (2006). Transjugular intrahepatic portosystemic shunt patency and clinical outcome in patients with Budd-Chiari syndrome: covered versus uncovered stents. *Radiology*.

[15] Sterling RK, Sanyal AJ (2000). Are TIPS tops in the treatment of portal hypertension? A review on the use and misuse of transjugular intrahepatic portosystemic shunts. *Canadian Journal of Gastroenterology*.

[16] Bambini DA, Superina R, Almond PS, Whitington PF, Alonso E (2000). Experience with the Rex shunt (mesenterico-left portal bypass) in children with extrahepatic portal hypertension. *Journal of Pediatric Surgery*.

[17] Dasgupta R, Roberts E, Superina RA, Kim PC (2006). Effectiveness of Rex shunt in the treatment of portal hypertension. *Journal of Pediatric Surgery*.

[18] Stringer MD (2007). Improved body mass index after mesenterico-portal bypass. *Pediatric Surgery International*.

[19] Superina R, Shneider B, Emre S, Sarin S, de Ville de Goyet J (2006). Surgical guidelines for the management of extra-hepatic portal vein obstruction. *Pediatric Transplantation*.

[20] Chin AC, Thow F, Superina RA (2008). Previous portal hypertension surgery negatively affects results of mesenteric to left portal vein bypass. *Journal of Pediatric Surgery*.

[21] Kim HB, Pomposelli JJ, Lillehei CW (2005). Mesogonadal shunts for extrahepatic portal vein thrombosis and variceal hemorrhage. *Liver Transplantation*.

